# Trends and determinants of stunting among under-5s: evidence from the 1995, 2001, 2006 and 2011 Uganda Demographic and Health Surveys

**DOI:** 10.1017/S1368980018001982

**Published:** 2018-08-29

**Authors:** Ying Ying Yang, Gabriella Kaddu, David Ngendahimana, Hope Barkoukis, Darcy Freedman, Yovani AM Lubaale, Ezekiel Mupere, Paul M Bakaki

**Affiliations:** 1 Hathaway Brown School, Shaker Heights, OH, USA; 2 Department of Biochemistry, Case Western Reserve University School of Medicine, Cleveland, OH, USA; 3 Department of Sociology, Case Western Reserve University College of Arts and Sciences, Cleveland, OH, USA; 4 Department of Population and Quantitative Health Sciences, Case Western Reserve University School of Medicine, 10900 Euclid Avenue, Cleveland, OH 44106, USA; 5 Department of Nutrition, Case Western Reserve University School of Medicine, Cleveland, OH, USA; 6 Department of Population Studies, School of Statistics and Planning, Makerere University, Kampala, Uganda; 7 Department of Pediatrics and Child Health, School of Medicine, Makerere University, Kampala, Uganda

**Keywords:** Trends, Undernutrition, Demographic and Health Surveys, Uganda, Stunting

## Abstract

**Objective:**

To describe trends of childhood stunting among under-5s in Uganda and to assess the impact of maternal education, wealth and residence on stunting.

**Design:**

Serial and pooled cross-sectional analyses of data from Uganda Demographic and Health Surveys (UDHS) of 1995, 2001, 2006 and 2011. Prevalence of stunting and mean height-for-age *Z*-score were computed by maternal education, wealth index, region and other sociodemographic characteristics. Multivariable logistic and linear regression models were fitted to survey-specific and pooled data to estimate independent associations between covariates and stunting or *Z*-score. Sampling weights were applied in all analyses.

**Setting:**

Uganda.

**Subjects:**

Children aged <5 years.

**Results:**

Weighted sample size was 14 747 children. Stunting prevalence decreased from 44·8% in 1995 to 33·2% in 2011. UDHS reported stunting as 38% in 1995, underestimating the decline because of transitioning from National Center for Health Statistics/Centers for Disease Control and Prevention standards to WHO standards. Nevertheless, one in three Ugandan children was still stunted by 2011. South Western, Mid Western, Kampala and East Central regions had highest odds of stunting. Being born in a poor or middle-income household, of a teen mother, without secondary education were associated with stunting. Other persistent stunting predictors included small birth size, male gender and age 2–3 years.

**Conclusions:**

Sustained decrease in stunting suggests that child nutrition interventions have been successful; however, current prevalence does not meet Millennium Development Goals. Stunting remains a public health concern and must be addressed. Customizing established measures such as female education and wealth creation while targeting the most vulnerable groups may further reduce childhood stunting.

Stunting, or inadequate height-for-age, is formally defined by the WHO as ‘the impaired growth and development that children experience from poor nutrition, repeated infection, and inadequate psychosocial stimulation’^(^ ^1^ ^)^. Stunting has adverse effects on children, including increased infectious disease morbidity and mortality, impaired cognitive and mental development, poor school performance, and low adult wages and productivity^(^ ^1^ ^,^ ^2^ ^)^. Overall, stunting negatively impacts the socio-economic development of a nation^(^ ^2^ ^)^.

Globally, it is estimated that stunting among children <5 years old (under-5s) decreased from 39·7 to 26·7% between 1990 and 2010^(^ ^3^ ^)^. During the same period, stunting in Africa stagnated around 40% and is expected to remain stable through 2020^(^ ^3^ ^)^. In Uganda, however, stunting decreased slowly over the same period. The reported prevalence of stunting was 38·3, 39·1, 38·1 and 33·4% for the years 1995, 2001, 2006 and 2011, respectively^(^ ^4^ ^)^. In 2011, it was estimated that 2·1 million Ugandan children were stunted.

There is strong evidence that sociodemographic variables are associated with stunting^(^ ^4^ ^–^ ^10^ ^)^, indicating that the prevalence at national level does not adequately represent stunting at regional level. The 2011 Uganda Demographic and Health Survey (UDHS) estimated that the Western region had the highest prevalence of stunting at 43·1%, followed by the Northern region with 32·2%. In Central and Eastern regions, stunting prevalence was 29·1 and 28·5%, respectively^(^ ^4^ ^)^. Apart from region, household factors such as wealth, source of drinking-water, toilet facility, number of household members^(^ ^5^ ^)^ and number of under-5 children^(^ ^6^ ^)^; maternal characteristics such as education, age^(^ ^5^ ^)^, height, marital status^(^ ^6^ ^,^ ^7^ ^)^, BMI^(^ ^6^ ^)^, work status^(^ ^8^ ^)^ and occupation^(^ ^7^ ^)^; and child-related characteristics including sex^(^ ^5^ ^,^ ^8^ ^)^, age^(^ ^9^ ^)^, birth order^(^ ^5^ ^,^ ^7^ ^)^, size at birth and recent morbidity^(^ ^9^ ^,^ ^10^ ^)^ are also associated with stunting.

The prevalence of stunting has been decreasing in Uganda, partly attributed to betterment of the economy and maternal education^(^ ^11^ ^)^. As an indicator for the improving economy, Uganda’s gross domestic product per capita grew from $280 in 1995 to $575 in 2011 in current US dollars^(^ ^12^ ^)^. Extreme poverty, defined as earning less than $1 per day for one individual, reduced from 56·4 % in 1992 to 24·5% in 2010^(^ ^11^ ^)^. In 1997, Uganda launched universal primary education, allowing all children to receive the first seven years of education for free^(^ ^11^ ^)^. Consequently, the percentage of women who did not receive formal education decreased from 31·0% in 1995^(^ ^13^ ^)^ to 19·9% in 2011^(^ ^4^ ^)^. The Government of Uganda partners with multiple international organizations to address childhood malnutrition and stunting. The Nutrition and Early Child Development Project^(^ ^14^ ^)^, with funds from the World Bank, was a multipronged nutrition intervention started in the late 1990s in two-thirds of the districts of Uganda selected based on prevalence of pre-school child malnutrition. UNICEF strives to ensure health care, nutrition and sanitation for children and mothers, who are reached by health-care workers from Village Health Teams in local facilities^(^ ^15^ ^)^. UNICEF also leads an Integrated Management of Acute Malnutrition plan to spread the correct child feeding practices and provide treatments for stunted children^(^ ^16^ ^)^. The Water, Sanitation, and Hygiene Programme was launched to increase access to clean water and sanitation^(^ ^16^ ^)^. In 2011, Uganda launched its multisectoral Uganda Nutrition Action Plan that specifically focuses on reducing malnutrition and the burden of mothers to care for their malnourished children^(^ ^17^ ^)^. In the same year, Uganda joined the Scaling Up Nutrition movement, a global movement that unites governments, non-government organizations and scholars to improve childhood nutrition^(^ ^17^ ^)^. Specifically, Scaling Up Nutrition coordinates with the implementation of the Uganda Nutrition Action Plan^(^ ^17^ ^)^.

Despite multiple efforts, Uganda still failed to meet the Millennium Development Goals proposed by the health ministry in 2005, which aimed to reduce stunting to 28 % by 2009^(^ ^18^ ^)^. As of 2011, one child in every three was stunted^(^ ^4^ ^)^. Strong surveillance systems are needed to guide strategies to achieve stunting reduction goals. While UDHS provides national comparisons of under-5 stunting in survey-specific publications, these data are limited in two ways. First, they do not offer longer-term trend analysis of stunting and its persistent risk factors needed by decision makers to act on. Second, national data do not offer key insights about regional variability in stunting, yet this level of granularity would allow for adjustment of strategies to target specific geographic areas with greater stunting burden. The aims of the present study were to describe the trends of under-5 childhood stunting in Uganda over 16 years and to assess its association with region of residence, maternal education and household income.

## Methods

### Study design

The present study was a serial cross-sectional analysis of four consecutive UDHS conducted in 1995, 2001, 2006 and 2011. UDHS are conducted nationwide every five years by the Uganda Bureau of Statistics to collect data on a wide range of topics including maternal and child health, household and respondent characteristics, education, nutrition and wealth.

### Study population

In 1995, 2001, 2006 and 2011, the number of households selected was 7550^(^ ^13^ ^)^, 7885^(^ ^19^ ^)^, 8870^(^ ^20^ ^)^ and 9033^(^ ^4^ ^)^, with response rates of 98·4, 95·8, 97·5 and 95·3%, respectively. The weighted sample size of under-5 children was 4738^(^ ^13^ ^)^, 5576^(^ ^19^ ^)^, 2378^(^ ^20^ ^)^ and 2055^(^ ^4^ ^)^ in survey years 1995, 2001, 2006 and 2011, respectively, resulting in an overall sample size of 14 747. Only about half (49·8%) of the sampled households had under-5 children, and the number of children per household decreased over surveys. UDHS studies are representative estimates for the country, region and rural–urban area. The samples were selected in two stages. In the first stage, enumeration areas were selected among all enumeration areas from the latest Uganda National Household Survey (UNHS) using the probability-proportional-to-size approach^(^ ^4^ ^,^ ^21^ ^,^ ^22^ ^)^. An enumeration area was a geographic area covering an average of 104 households for survey year 2011^(^ ^4^ ^)^, while the sizes of enumeration areas in previous surveys were not included in the UDHS reports. In the second stage, a fixed number of households from each enumeration area was randomly selected. The second stage compensated the first stage, so that each individual in the population had the same probability of being sampled. All women aged 15–49 years who were resident in the selected households were eligible for interview. Height and weight measurements were carried out on women aged 15–49 years and their under-5 children.

UDHS data were anonymous regarding participant identities such as name and address. The institutional review boards determined that the present study was not a human subjects study (see online supplementary material, Supplemental Fig. 1). UDHS data were accessible upon approval by the DHS office located in Washington, DC.

**Fig. 1:**
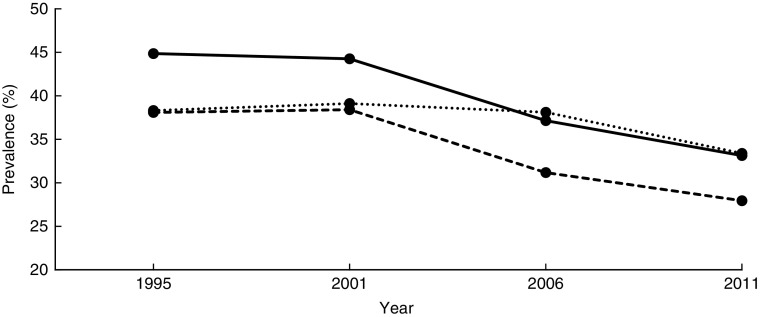
Overall stunting trends among children aged <5 years according to standard (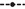, National Center for Health Statistics/Centers for Disease Control and Prevention International Reference Population^(^ ^24^ ^)^; 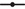, WHO Child Growth Standards^(^ ^23^ ^)^; 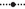, Uganda Demographic and Health Survey published data): Uganda Demographic and Health Survey 1995, 2001, 2006 and 2011^(^ ^4^ ^,^ ^13^ ^,^ ^19^ ^,^ ^20^ ^)^

### Variables of interest

#### Outcome variables: stunting as a binary variable and height-for-age Z-score

In the present study, stunting was measured by height-for-age *Z*-score (HAZ) using the WHO Child Growth Standards. In our primary analysis, a child was moderately to severely stunted if his or her height-for-age was below −2 sd of the median of the WHO standard (i.e. HAZ<–2)^(^ ^23^ ^)^. To assess the robustness of our findings, severe stunting (height-for-age below −3 sd of the median of the WHO standard; i.e. HAZ<–3) was used in sensitivity analyses for comparison with the primary analysis. In addition to measuring stunting as a binary variable, HAZ was examined as a continuous outcome variable in sensitivity analyses. HAZ according to both the US National Center for Health Statistics/Centers for Disease Control and Prevention (NCHS/CDC) International Reference Population^(^ ^24^ ^)^ and the WHO Child Growth Standards were available in the DHS data. The UDHS reported stunting based on the NCHS/CDC reference in 1995 and 2001, and according to the WHO standards in 2006 and 2011. We plotted and compared the prevalence of stunting based on the NCHS/CDC reference and WHO standards for all surveys with the UDHS reported prevalence, to assess the effect of migration from the NCHS/CDC reference to WHO standards on the published trends of stunting. Additionally, we fitted a logistic regression model to the NCHS/CDC reference-based stunting data as part of sensitivity analyses to assess if the same risk factors held in both data sets.

#### Predictor variables: geographical region, household wealth index and maternal education

Regions were collapsed from the ten regions in recent surveys to the four regions in earlier surveys for comparison over survey years: Northern (West Nile, Mid Northern and North East), Central (Central 1, Central 2 and Kampala), Eastern (East Central and Mid Eastern) and Western (South Western and Mid Western). In a sub-analysis of 2006 and 2011 surveys, nine sub-regions were used to evaluate their association with stunting (see online supplementary material, Supplemental Fig. 2). The wealth index, calculated and provided by the UDHS, used assets to approximate wealth of households (Supplemental Table 1). The household wealth index was available from the data set in quintiles labelled as: poorest, poor, middle, rich and richest^(^ ^4^ ^)^. It was computed by the UDHS using factor analysis. Maternal education was categorized as none, primary (1–7 years), and secondary or higher (≥8 years). Discrete years of school were used for exploratory analysis.

**Fig. 2:**
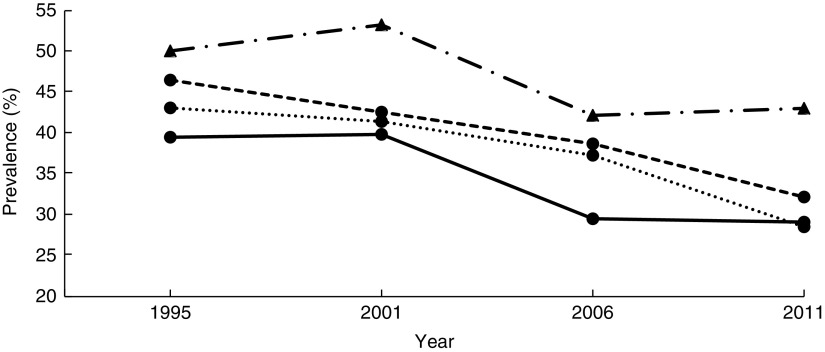
Stunting trends, based on the WHO Child Growth Standards^(^ ^23^ ^)^, among children aged<5 years according to region (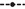, Central; 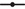, Eastern; 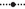, Northern; 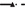, Western): Uganda Demographic and Health Survey 1995, 2001, 2006 and 2011^(^ ^4^ ^,^ ^13^ ^,^ ^19^ ^,^ ^20^ ^)^

**Table 1:** Distribution of household, maternal and child-related characteristics: Uganda Demographic and Health Survey 1995, 2001, 2006 and 2011^(^ ^4^ ^,^ ^13^ ^,^ ^19^ ^,^ ^20^ ^)^

	1995 (*n* 4738)		2001 (*n* 5576)		2006 (*n* 2378)		2011 (*n* 2055)		Overall (*n* 14 747)	
	*n*	%	*n*	%	*n*	%	*n*	%	*n*	%
Household characteristics										
Region										
Central	1218	25·7	1480	26·5	561	23·6	493	24·0	3752	25·4
Eastern	1264	26·7	1721	30·9	637	26·8	646	31·4	4268	28·9
Northern	917	19·3	962	17·3	514	21·6	379	18·4	2772	18·8
Western	1339	28·3	1413	25·3	666	28·0	538	26·2	3956	26·8
Residence										
Urban	533	11·3	534	9·6	257	10·8	281	13·7	1605	10·9
Rural	4204	88·7	5042	90·4	2122	89·2	1774	86·3	13142	89·1
Source of drinking-water										
Improved	2275	48·1	2313	41·7	1031	43·6	1251	60·9	6870	46·8
Non-improved	2451	51·9	3230	58·3	1332	56·4	804	39·1	7817	53·2
Toilet facility										
Improved	3836	81·2	4442	79·8	1244	52·3	993	48·4	10514	71·4
Non-improved	887	18·8	1121	20·2	1135	47·7	1060	51·6	4203	28·6
No. of household members										
1–5	1878	39·6	2307	41·4	816	34·3	823	40·0	5824	39·5
5–9	2197	46·4	2543	45·6	1240	52·1	978	47·6	6959	47·2
≥10	662	14·0	726	13·0	322	13·6	254	12·4	1965	13·3
No. of under-5 children in household										
1	1173	24·8	1355	24·3	557	23·4	472	23·0	3557	24·1
2	2040	43·1	2532	45·4	1121	47·1	956	46·5	6649	45·1
≥3	1524	32·2	1689	30·3	700	29·4	627	30·5	4541	30·8
Maternal characteristics										
Age (years)										
15–19	486	10·3	343	6·2	107	4·5	117	5·7	1053	7·1
20–30	2915	61·5	3462	62·1	1359	57·1	1187	57·8	8923	60·5
31–49	1336	28·2	1771	31·8	913	38·4	751	36·5	4771	32·3
BMI/pregnancy status										
Pregnant/postpartum	861	18·3	1349	24·4	592	24·9	476	23·3	3278	22·4
Thin	336	7·1	338	6·1	201	8·5	166	8·1	1040	7·1
Normal	3031	64·5	3166	57·4	1234	52·0	1046	51·1	8477	57·9
Overweight/obese	470	10·0	666	12·1	347	14·6	359	17·5	1842	12·6
Marital status										
Single	515	10·9	590	10·6	271	11·4	234	11·4	1610	10·9
Married	4222	89·1	4985	89·4	2108	88·6	1821	88·6	13137	89·1
Highest educational level										
None	1467	31·0	1361	24·4	525	22·1	253	12·3	3606	24·5
Primary	2748	58·0	3589	64·4	1525	64·1	1352	65·8	9214	62·5
Secondary or higher	523	11·0	626	11·2	329	13·8	450	21·9	1927	13·1
Work status										
Unemployed	1634	34·7	862	15·5	178	7·5	436	21·2	3110	21·1
Self-employed	2371	50·3	4011	72·0	1741	73·3	1086	52·9	9209	62·6
Employed	711	15·1	699	12·5	456	19·2	532	25·9	2398	16·3
Child-related characteristics										
Sex										
Male	2310	48·8	2769	49·7	1207	50·8	1024	49·8	7310	49·6
Female	2428	51·2	2807	50·3	1171	49·2	1032	50·2	7437	50·4
Current age (months)										
0–12	1482	31·3	1294	23·2	566	23·8	483	23·5	3825	25·9
13–23	1302	27·5	1233	22·1	466	19·6	440	21·4	3440	23·3
24–35	999	21·1	1036	18·6	480	20·2	387	18·8	2901	19·7
36–59	955	20·1	2013	36·1	867	36·4	746	36·3	4581	31·1
Low birth size										
Yes	898	19·2	1040	18·9	465	19·6	559	22·1	2846	19·5
No	3785	80·8	4469	81·1	1900	80·4	4431	77·9	11712	80·5
Birth order										
1	899	19·0	888	15·9	359	15·1	332	16·1	2478	16·8
2–3	1540	32·5	1825	32·7	679	28·6	666	32·4	4710	31·9
4–5	1015	21·4	1316	23·6	572	24·1	454	22·1	3358	22·8
≥6	1283	27·1	1547	27·7	768	32·3	604	29·4	4202	28·5
Preceding birth interval less than 24 months										
Yes	958	20·2	1215	21·8	468	19·7	417	20·3	3058	20·7
No	3776	79·8	4356	78·2	1910	80·3	1637	79·7	11679	79·3
Had fever in last two weeks										
Yes	2296	48·7	2614	47·0	1042	43·9	911	44·3	6863	46·6
No	2421	51·3	2953	53·0	1333	56·1	1144	55·7	7851	53·4
Had cough in last two weeks										
Yes	2431	51·4	2743	49·3	1156	48·7	1025	49·9	7355	49·9
No	2299	48·6	2824	50·7	1220	51·3	1030	50·1	7373	50·1
Had diarrhoea in last two weeks										
Yes	1170	24·8	1171	21·0	645	27·2	507	24·7	3493	23·7
No	3556	75·2	4391	79·0	1728	72·8	1548	75·3	11223	76·3

#### Household, maternal and child-related covariates

Household characteristics included urban–rural residence, source of drinking-water (improved *v.* non-improved), toilet facility (improved *v.* non-improved), number of household members and number of under-5 children in the household. Maternal characteristics included maternal age, marital status, BMI/pregnancy status and work status. The child-related characteristics included sex, age, birth order, preceding birth interval less than 24 months, size at birth, fever in the last two weeks, cough in the last two weeks and diarrhoea in the last two weeks. Size at birth was subjectively reported by the respondent, and we categorized smaller than average and very small as low birth size as done in other studies^(^ ^25^ ^,^ ^26^ ^)^.

### Data analysis

The data were analysed using the statistical software package SAS version 9.4. The initial analysis involved univariable and bivariable analyses using *χ* ^2^ tests to assess associations between categorical variables and stunting for the primary analysis. All predictor variables were categorical, the moderate to severe stunting primary outcome variable and the severe stunting outcome variable were binary, while the HAZ outcome variable was continuous. Variables that were associated with stunting in the bivariable analysis at a *P* value of 0·1 were included in multivariable logistic and linear regression models to assess their independent associations with stunting and HAZ, respectively. For each survey, a model was fitted to the data, and thereafter the data were pooled across all surveys to fit an overall model. The survey indicator variable was included in the pooled models as a dummy variable to account for temporal variation and direction of the strength of the OR. Backward elimination of non-significant variables (at a *P* value of ≥0·05) was performed to obtain a preliminary model for each survey. Variables were retained in all final models if they were statistically associated with stunting at a *P* value of <0·05 in any of the survey-specific models. To account for the DHS complex sampling design, sampling weights, provided by UDHS, were applied in all analyses. Sensitivity analyses were performed in a similar way as the primary analysis except that we used ANOVA for the bivariable analysis of HAZ.

## Results

### Overall decreasing trends of stunting

The present study included under-5 children from four UDHS. The sample size was 4738, 5576, 2378 and 2055 for surveys conducted in 1995, 2001, 2006 and 2011, respectively. Overall, children from the Northern region constituted the smallest proportion of 19 %; children from each of the other three regions constituted more than one-quarter of the sample. Almost 90 % of the children were rural residents. About 59 % of the children were aged <24 months in the 1995 survey. This proportion dropped to 45% in 2001 and stayed about the same in subsequent surveys. In the first three surveys, about 19 % of the children were considered small at birth by their mothers, which proportion increased to 22·1 % in 2011. Morbidity within two weeks of data collection was high in all surveys: fever, 44–49 %; cough, 49–51 %; and diarrhoea, 21–27 % (Table 1).

The mean HAZ increased from −1·8 (se 0·027) in 1995 to −1·4 (se 0·039) in 2011 (Table 2). The prevalence of stunting decreased from 44·8 to 33·2% over the same period (*χ* ^2^ for trend=4·5, *P* < 0·0001). Figure 1 shows the prevalence of stunting computed according to the NCHS/CDC reference and the WHO standards, and that reported by the UDHS, which masks the sharp drop in stunting between 2001 and 2006. A 7-percentage-point reduction was masked in the UDHS reports due to migration from the NCHS/CDC reference to the WHO standards in 2006. The NCHS/CDC reference underestimated stunting at 38·3% instead of 44·8 % in 1995 and at 39·1% instead of 44·2% in 2001. If the NCHS/CDC reference were used in 2006 and 2011, stunting would have been reported, respectively, as 31·2% instead of 38·1% and 28·0% instead of 33·4%. Using year 1995 as a reference, the adjusted OR (AOR) of stunting in years 2001, 2006 and 2011 was 0·89 (95% CI 0·81, 0·99; *P*<0·05), 0·65 (95% CI 0·57, 0·73; *P*<0·001) and 0·58 (95% CI 0·51, 0·67; *P*<0·001), respectively (Table 3). This was a 42% reduction in in the odds of stunting over 16 years.

**Table 2:** Number and percentage of children aged<5 years with stunting, based on the WHO Child Growth Standards^(^ ^23^ ^)^, by household, maternal and child-related characteristics: Uganda Demographic and Health Survey 1995, 2001, 2006 and 2011^(^ ^4^ ^,^ ^13^ ^,^ ^19^ ^,^ ^20^ ^)^

	1995 (*n* 4738)		2001 (*n* 5576)		2006 (*n* 2378)		2011 (*n* 2055)	
	*n*	%	*n*	%	*n*	%	*n*	%
Overall HAZ								
Mean	−1·8		−1·8		−1·5		−1·4	
se	0·027		0·023		0·035		0·039	
Overall stunted	2123	44·8	2466	44·2	883	37·1	681	33·2
Region		***		***		***		***
Central	481	39·5	590	39·9	166	29·5	144	29·1
Eastern	545	43·1	713	41·4	238	37·3	184	28·5
Northern	426	46·5	410	42·6	199	38·7	122	32·2
Western	671	50·1	753	53·3	281	42·2	232	43·1
Residence		***		***		***		***
Urban	147	27·6	168	31·4	67	25·9	52	18·7
Rural	1976	47·0	2298	45·6	817	38·5	629	35·4
Wealth index		***		***		***		***
Poorest	521	50·2	615	49·2	211	42·4	159	35·7
Poorer	505	51·4	577	46·8	184	35·7	136	30·5
Middle	428	44·2	568	47·9	227	43·7	191	45·7
Richer	387	44·2	458	42·8	163	37·0	118	30·3
Richest	282	32·3	247	29·5	98	24·2	77	21·7
Source of drinking-water		***				***		***
Improved	940	41·3	1051	45·4	333	32·3	375	30·0
Non-improved	1177	48·0	1398	43·3	542	40·7	306	38·1
Toilet facility								
Improved	1692	44·1	1961	44·1	468	37·7	337	34·0
Non-improved	424	47·8	498	44·4	415	36·6	344	32·5
No. of household members								
1–5	845	45·0	1038	45·0	293	35·9	261	31·8
5–9	297	44·8	1132	44·5	478	38·6	332	33·9
≥10	981	44·7	296	40·7	113	34·9	88	34·7
No. of under-five children in household								*
1	514	43·8	586	43·2	187	33·7	138	29·2
2	902	44·2	1110	43·8	424	37·8	310	32·4
≥3	707	46·4	770	45·6	272	38·8	233	37·2
Mother’s age (years)								
15–19	209	43·0	152	44·4	40	37·6	46	39·0
20–30	1336	45·8	1529	44·2	508	37·4	404	34·1
31–49	578	43·2	784	44·3	335	36·7	231	30·8
BMI/pregnancy status		***		**		*		**
Pregnant/postpartum	427	49·6	624	46·3	231	39·0	185	38·8
Thin	174	51·7	158	46·7	72	35·7	50	29·9
Normal	1344	443	1413	44·6	479	38·8	358	34·2
Overweight/obese	161	34·2	247	37·2	99	28·5	89	24·8
Marital status								
Single	224	43·5	272	46·1	108	39·8	74	31·5
Married	1899	45·0	2194	44·0	776	36·8	608	33·4
Highest educational level		***		***		***		***
None	704	48·0	707	51·9	214	40·8	101	39·8
Primary	1244	45·3	1548	43·1	595	39·0	467	34·7
Secondary or higher	175	33·4	211	33·7	74	22·5	111	24·7
Work status		***		***		**		***
Unemployed	709	43·4	340	39·5	59	33·2	120	27·4
Self-employed	1156	48·8	1881	46·9	686	39·4	410	37·7
Employed	246	34·6	243	34·8	138	30·3	152	28·6
Sex		***		***		**		***
Male	1122	48·6	1300	47·0	485	40·2	381	37·3
Female	1001	41·2	1165	41·5	398	34·0	300	29·1
Child’s current age (months)		***		***		***		***
0–12	378	25·5	313	24·2	110	19·5	79	16·3
13–23	643	49·4	614	49·8	194	41·7	168	38·1
24–35	572	57·2	562	54·3	224	46·7	175	45·3
36–59	530	55·5	976	48·5	355	41·0	260	34·9
Low birth size		***		***		***		***
Yes	466	51·9	532	51·2	224	48·2	189	42·6
No	1632	43·1	1904	42·6	654	34·4	471	30·2
Birth order								
1	416	46·3	390	43·9	132	36·7	114	34·4
2–3	682	44·3	797	43·7	258	38·0	218	32·7
4–5	438	43·2	594	45·1	208	36·3	139	30·5
≥6	586	45·7	685	44·2	286	37·2	211	34·9
Preceding birth interval less than 24 months				*				
Yes	449	46·8	582	47·9	191	40·8	157	37·7
No	1672	44·3	1881	43·2	692	36·2	524	32·0
Had fever in last two weeks		**						
Yes	1085	47·2	1188	45·4	397	38·1	304	33·4
No	1028	42·5	1272	43·1	486	36·4	377	33·0
Had cough in the last two weeks								
Yes	1123	46·2	1234	45·0	450	38·9	353	34·5
No	996	43·3	1226	43·4	433	35·5	328	31·8
Had diarrhoea in the last two weeks		**				**		
Yes	581	49·6	551	47·1	272	42·2	176	34·6
No	1538	43·3	1907	43·4	611	35·3	506	32·7

HAZ, height-for-age *Z*-score. *P≤0·05, **P≤0·01, ***P≤0·001.

**Table 3:** Relationship between childhood stunting, based on the WHO Child Growth Standards^(^ ^23^ ^)^, and household, maternal and child-related characteristics from the multivariable logistic regression models: Uganda Demographic and Health Survey 1995, 2001, 2006 and 2011^(^ ^4^ ^,^ ^13^ ^,^ ^19^ ^,^ ^20^ ^)^

	1995		2001		2006		2011		Overall	
	OR	95% CI	OR	95% CI	OR	95% CI	OR	95% CI	OR	95% CI
Household characteristics										
Region										
Central	1·24	1·00, 1·54	1·28	1·05, 1·55*	0·84	0·61, 1·16	1·51	1·03, 2·22*	1·25	1·11, 1·42***
Northern	1·06	0·81, 1·39	0·92	0·74, 1·13	1·00	0·75, 1·33	1·17	0·81, 1·69	1·02	0·89, 1·16
Western	1·60	1·29, 1·97***	1·76	1·46, 2·12***	1·27	0·95, 1·70	2·15	1·49, 3·10***	1·74	1·55, 1·96***
Eastern (ref.)	1·00	–	1·00	–	1·00	–	1·00	–	1·00	–
Residence										
Rural	1·59	1·25, 2·02***	0·95	0·74, 1·23	0·96	0·62, 1·48	1·36	0·84, 2·23	1·20	1·03, 1·40*
Urban (ref.)	1·00	–	1·00	–	1·00	–	1·00	–	1·00	–
Wealth index										
Poorest	1·53	1·13, 2·06**	2·17	1·61, 2·91***	1·60	1·04, 2·45*	1·28	0·73, 2·24	1·73	1·45, 2·06***
Poorer	1·53	1·15, 2·03**	1·92	1·43, 2·57***	1·14	0·76, 1·72	1·02	0·59, 1·75	1·50	1·26, 1·78***
Middle	1·13	0·85, 1·49	1·99	1·50, 2·63***	1·66	1·13, 2·45*	2·11	1·26, 3·53**	1·61	1·36, 1·90***
Richer	1·20	0·91, 1·57	1·66	1·26, 2·18***	1·37	0·94, 2·01	1·15	0·68, 1·92	1·37	1·16, 1·61***
Richest (ref.)	1·00	–	1·00	–	1·00	–	1·00	–	1·00	–
Source of drinking-water										
Non-improved	1·11	0·95, 1·30	0·96	0·84, 1·11	1·30	1·05, 1·61*	1·32	1·01, 1·72*	1·09	1·00, 1·18
Improved (ref.)	1·00	–	1·00	–	1·00	–	1·00	–	1·00	–
Maternal characteristics										
Age (years)										
15–19	1·46	1·10, 1·95**	1·53	1·14, 2·04**	1·58	0·96, 2·60	2·21	1·29, 3·81**	1·57	1·31, 1·87***
20–30	1·28	1·08, 1·52**	1·10	0·95, 1·28	1·12	0·91, 1·37	1·32	1·03, 1·70*	1·18	1·08, 1·29***
31–49 (ref.)	1·00	–	1·00	–	1·00	–	1·00	–	1·00	–
BMI/pregnancy status										
Pregnant/postpartum	1·01	0·83, 1·23	1·02	0·86, 1·21	0·94	0·75, 1·19	1·46	1·08, 1·97*	1·04	0·94, 1·16
Thin	1·33	0·99, 1·77	1·10	0·83, 1·46	0·83	0·57, 1·21	0·90	0·59, 1·39	1·08	0·92, 1·27
Overweight/obese	0·67	0·52, 0·86**	0·76	0·61, 0·93**	0·64	0·46, 0·88**	0·68	0·47, 0·99*	0·70	0·61, 0·79***
Normal (ref.)	1·00	–	1·00	–	1·00	–	1·00	–	1·00	–
Highest educational level										
None	1·22	0·91, 1·62	1·36	1·03, 1·80*	1·58	1·06, 2·35*	1·54	0·95, 2·52	1·34	1·14, 1·59***
Primary	1·24	0·96, 1·60	1·01	0·79, 1·30	1·69	1·20, 2·38***	1·29	0·88, 1·87	1·20	1·04, 1·38*
Secondary or higher (ref.)	1·00	–	1·00	–	1·00	–	1·00	–	1·00	–
Work status										
Unemployed	1·16	0·92, 1·48	1·07	0·83, 1·38	1·38	0·89, 2·14	0·79	0·54, 1·16	1·06	0·92, 1·23
Self-employed	1·28	1·01, 1·63*	1·21	0·96, 1·53	1·07	0·80, 1·42	1·08	0·78, 1·50	1·18	1·04, 1·34*
Employed (ref.)	1·00	–	1·00	–	1·00	–	1·00	–	1·00	–
Child-related characteristics										
Sex										
Male	1·44	1·24, 1·67***	1·31	1·15, 1·50***	1·46	1·20, 1·78***	1·72	1·35, 2·20***	1·42	1·31, 1·54***
Female (ref.)	1·00	–	1·00	–	1·00	–	1·00	–	1·00	–
Current age (months)										
13–23	3·07	2·51, 3·75***	3·32	2·70, 4·08***	3·31	2·40, 4·58***	3·96	2·69, 5·85***	3·22	2·85, 3·65***
24–35	4·76	3·83, 5·91***	4·48	3·59, 5·60***	4·24	3·09, 5·83***	5·68	3·80, 8·50***	4·61	4·05, 5·25***
36–59	4·69	3·76, 5·85***	3·58	2·94, 4·37***	3·49	2·61, 4·67***	3·66	2·53, 5·31***	3·75	3·32, 4·23***
0–12 (ref.)	1·00	–	1·00	–	1·00	–	1·00	–	1·00	–
Low birth size										
Yes	1·44	1·19, 1·74***	1·50	1·26, 1·79***	1·89	1·48, 2·41***	1·99	1·50, 2·64***	1·59	1·44, 1·77***
No (ref.)	1·00	–	1·00	–	1·00	–	1·00	–	1·00	–
Preceding birth interval less than 24 months										
Yes	1·11	0·93, 1·32	1·25	1·06, 1·47**	1·23	0·95, 1·57	1·23	0·92, 1·63	1·19	1·08, 1·32***
No (ref.)	1·00	–	1·00	–	1·00	–	1·00	–	1·00	–
Had fever in last two weeks										
Yes	1·18	1·01, 1·38*	1·09	0·95, 1·25	0·94	0·76, 1·15	1·11	0·85,1·44	1·08	0·99, 1·18
No (ref.)	1·00	–	1·00	–	1·00	–	1·00	–	1·00	–
Had diarrhoea in last two weeks										
Yes	1·17	1·07, 1·28***	1·17	1·07, 1·27***	1·23	1·10, 1·39***	1·11	0·97,1·28	1·17	1·11, 1·23***
No (ref.)	1·00	–	1·00	–	1·00	–	1·00	–	1·00	–
Survey year										
2001									0·89	0·81, 0·99*
2006									0·64	0·57, 0·73***
2011									0·58	0·51, 0·67***
1995 (ref.)									1·00	–

ref., reference category. *P≤0·05, **P≤0·01, ***P≤0·001. OR are adjusted in multivariable models. All variables in Table 3 are included in each model.

#### Household characteristics: region, residence, wealth index and source of drinking-water

In Eastern and Northern Uganda, the prevalence of stunting declined steadily, consistent with the trend at the national level. Stunting in Central Uganda stagnated from 1995 to 2001, while the prevalence in Western Uganda increased by 3·2 percentage points. Between 2001 and 2006, Central and Western Uganda experienced a sharp drop, but stunting prevalence stagnated thereafter (39·5, 39·9, 29·5 and 29·1% in Central; 50·1, 53·3, 42·2 and 43·1% in Western for years 1995, 2001, 2006, 2011, respectively; Table 2 and Fig. 2). Compared with children from Eastern Uganda, the pooled AOR was 1·25 (95% CI 1·11, 1·42) in Central and 1·74 (95% CI 1·55, 1·95) in Western (*P*<0·001 for both regions; Table 3). Sub-analysis of 2006 and 2011 data revealed that children from the South Western sub-region had the highest odds of stunting among all nine sub-regions (AOR=2·23; 95% CI 1·63, 3·06; *P*<0·001), using Mid Eastern sub-region as the reference (see online supplementary material, Supplemental Table 2). Other sub-regions with significantly higher odds of stunting than the Mid Eastern sub-region were Kampala (AOR=1·69; 95% CI 1·14, 2·50; *P*<0·001), East Central (AOR=1·42; 95% CI 1·06, 1·90; *P*<0·01) and Western (AOR=1·65; 95% CI 1·22, 2·23; *P*<0·001). The proportion of stunting was higher in rural areas than in urban areas in all surveys (Table 2), but only in year 1995 did rural children have significantly higher relative odds (AOR=1·59; 95 % CI 1·25, 2·02; *P*<0·001) compared with urban children. Overall, rural children were 20 % more likely to be stunted when compared with urban children (Table 3).

When grouping households by wealth index quintiles, the richest quintile had the lowest proportion of stunting in every survey (Table 2). From 1995 to 2011, the proportion of stunting in the middle quintile of the wealth index stagnated at about 45 %, while the proportion of stunting in the other four quintiles declined steadily (Table 2). Using the richest quintile as a reference, children in the poorest and poorer quintiles had significantly higher odds of stunting (AOR=1·53; 95% CI 1·13, 2·06; *P*<0·01 and AOR=1·53; 95% CI 1·15, 2·03; *P*<0·01, respectively) in 1995. This phenomenon shifted as, in 2011, only the middle quintile had significantly higher odds of stunting (AOR=2·11; 95% CI 1·26, 3·53; *P*<0·01; Table 3). Availability of improved source of drinking-water was about 40 % in the first three surveys but increased to more than 60 % in 2011 (Table 1). In 2006 and 2011, children who used non-improved water sources were significantly more likely to be stunted that those who used improved water sources.

### Maternal characteristics: education, age, BMI and employment

In each survey, women had greater access to education than the survey before. The prevalence of women with no education declined steadily from 31·0% in 1995 to 12·3% 2011 (*χ* ^2^ for trend=8·4, *P*<0·0001). Women with primary and secondary or higher education increased from 58·0 to 65·8 % and from 11·0 to 21·9 % over the same period. In all surveys, the proportion of stunting in children declined as mother’s educational level increased. Using mothers with secondary or higher education as the reference group, children of uneducated or primary educated mothers were significantly more likely to be stunted in the pooled model (AOR=1·34; 95% CI 1·14, 1·59; *P*<0·001 and AOR=1·20; 95% CI 1·04, 1·38; *P*<0·05, respectively). Similar associations were observed in the 2001 and 2006 surveys but not in the 1995 or 2011 surveys (Table 3). Finer analysis of years of school revealed stepladder reduction in child stunting if the mother stopped in senior one (eighth year of education) or senior five (twelfth year of education), coinciding with successful completion of primary and secondary education, respectively (see online supplementary material, Supplemental Table 3).

Throughout all four surveys, 20–30-year-old mothers were the dominant age group, and the proportion of teen mothers almost halved between 1995 and 2001 (Table 1). Childhood stunting decreased in all maternal age groups, but the decline was lowest for children with teenage mothers (Table 2). There was an increased likelihood of childhood stunting if the mother was younger (AOR=1·57; 95% CI 1·31, 1·87; *P*<0·001; Table 3). When categorizing women by maternal BMI/pregnancy status, stunting was most prevalent in children who had pregnant or postpartum mothers, defined as the first two months after delivery. The prevalence of stunting decreased most among children with thin mothers (Table 2). Overweight or obese mothers were significantly less likely to have stunted children in all four surveys compared with mothers of normal BMI (AOR=0·70; 95 % CI 0·61, 0·79; *P*<0·001; Table 3). Other than survey year 1995, there was no significant association between maternal employment status and child stunting after adjusting for other variables.

### Child-related characteristics: sex, age, birth weight and diarrhoea

In each survey and the pooled model, male children were significantly more likely to be stunted than female children (AOR=1·42; 95% CI 1·31, 1·54; *P*<0·001; Table 3). Children aged 24–35 months were the most stunted, while those aged 0–12 months were least stunted (Table 2). Prevalence of stunting was significantly higher among children who were small at birth than among those with normal birth size. Over the period of study, stunting among children with normal birth size decreased by 13 percentage points compared with a 9-percentage-point decrease among children with small size (Table 2). A child with diarrhoea two weeks before the survey was 17% more likely to be stunted than one without (AOR=1·17; 95% CI 1·11, 1·23; *P*<0·001). Similar associations were observed in survey-specific estimates (Table 3). When severe stunting (HAZ<−3) or continuous HAZ was the outcome variable or the NCHS/CDC reference were used in sensitivity analyses, the findings were similar to those reported when moderate to severe stunting (HAZ<−2) was the outcome variable (see online supplementary material, Supplemental Table 4).

## Discussion

The present study examined the long-term trends of stunting among under-5 Ugandan children between 1995 and 2011 by various characteristics. At the national level, childhood stunting decreased significantly from 44·8% in 1995 to 33·2% in 2011 using the WHO child growth standards. However, a 7-percentage-point reduction in stunting was masked in the UDHS reports due to migration from the NCHS/CDC reference to the WHO standards in 2006. Geographical region, household wealth, maternal education and child age, gender and birth size were associated with childhood stunting over the four surveys. These findings held when using the WHO standards or NCHS/CDC reference, or continuous HAZ.

Similar studies showed a slower decline of childhood stunting in Kenya and Côte d’Ivoire, where the prevalence of stunting decreased by 4·6 and 2·9 percentage points in the past two decades, respectively^(^ ^6^ ^,^ ^27^ ^)^. It is not clear whether transition from the NCHS/CDC reference to the WHO standards partially explains the minimal decreases in stunting in these countries. High proportions of stunting among children were also found in the Central African Republic, Democratic Republic of Congo and Ethiopia^(^ ^28^ ^–^ ^30^ ^)^. Compared with the whole of sub-Saharan Africa, where 40% of under-5 children were stunted^(^ ^3^ ^)^, Uganda is performing better. However, Uganda failed to meet its goal of reducing stunting to 28% by 2009^(^ ^18^ ^)^. Regional differences in the prevalence of stunting were found in similar studies conducted in Tanzania, Nigeria, Kenya and Zambia^(^ ^31^ ^–^ ^33^ ^)^. Western Uganda had the highest prevalence of stunting, which could be attributed to incorrect feeding practices, local dietary traditions and HIV/AIDS^(^ ^34^ ^–^ ^36^ ^)^. UNICEF and WHO recommend exclusive breast-feeding for the first 6 months of life and introduction of complementary foods at 6 months in addition to continued breast milk^(^ ^4^ ^)^. However, a study in Western Uganda found that 21% of 2–3-month-old infants received complementary foods^(^ ^34^ ^)^. Moreover, the frequency of complementary feeding for almost half of the children aged 12–23 months was less than optimal (less than twice per day)^(^ ^34^ ^)^. Local diets may also contribute to stunting. Western Uganda has high agricultural potential as vegetables can be grown year-round, however they are not widely consumed^(^ ^36^ ^)^. Goode noted that popularizing the consumption of plants would improve nutrient intakes as vegetables provide micronutrients such as vitamin A, iron and zinc^(^ ^36^ ^)^. Furthermore, HIV/AIDS is associated with lower health status of parents, which is associated with increased childhood stunting^(^ ^35^ ^)^. In Western Uganda, the higher childhood malnutrition rate coincides with higher HIV infection rate^(^ ^35^ ^)^. A sub-analysis of the 2006 and 2011 surveys including nine smaller regions revealed small regional variation in stunting, warranting smaller area analysis to pinpoint pockets of stunting. Policies regarding improving children’s diets need to be made, targeting the Western region and other smaller regions with the highest prevalence of stunting.

While it is not logical to compare household wealth index quintiles across surveys because the cut-offs may be different, comparing yearly prevalence of stunting in different quintiles helps to visualize the disparity of nutritional status among children from different economic levels. Using the richest category as reference, children from the poorest and poorer categories were significantly more likely to be stunted in 1995. Similar results were found in Tanzania, Nigeria, Kenya and Zambia^(^ ^31^ ^–^ ^33^ ^)^, where children from poorer families were more likely to be stunted. One explanation is that richer households would have greater purchasing power to ensure adequate nutritional intake for children compared with poorer households^(^ ^2^ ^)^. A shift in the group most vulnerable to stunting regarding wealth index was observed in later survey years. In 2001, while all four groups had significantly higher odds of stunting when compared with the richest group, the poorest and middle categories had the highest OR. In 2011, only children from the middle category were significantly more likely to be stunted with a twofold increase. It is not clear why the children most vulnerable to stunting shifted from the poorest households to the middle-income households over time. This observation needs to be researched further. Rural–urban migration may partially explain this observation as household income may be spent on asset acquisition in preference to buying food; commercialization of food crops that came with economic diversification during our study period may have led to families selling food to acquire assets instead of eating it^(^ ^37^ ^)^. It is also possible that household assets that correlate with household income changed over the study period and need re-evaluation^(^ ^38^ ^,^ ^39^ ^)^.

Consistent with the UDHS reports and previous research in Uganda^(^ ^9^ ^)^, higher maternal education was associated with lower likelihood of childhood stunting^(^ ^4^ ^)^. A closer look at completed years of maternal education revealed that reaching senior one (or finishing the 7-year primary education) or senior five (or finishing the 4-year lower secondary education) was associated with the highest reduction in stunting of children, suggesting that successful graduation from primary or secondary school is more beneficial than simply accumulating years of education. This is consistent with a Kenyan study which found that attaining a secondary level of maternal education was associated with decreased odds of childhood stunting, since higher education is associated with employment and higher household income^(^ ^6^ ^)^. Education also serves as an important strategy to teach women correct child feeding practices^(^ ^40^ ^)^. Unfortunately, as of 2011, 12% of women in Uganda remained uneducated, while 66% received only some primary education. Only 22% received some secondary education or higher. Structural inequalities may keep women out of school, as many African cultures consider men’s education more important than the education of women^(^ ^41^ ^)^. According to the Uganda Bureau of Statistics, of all females aged 6–12 years who did not attend school, 53·8% did not attend because they were considered to be ‘too young’; another 6% did not go to school because they had to help at home, while only 2·6% of their male counterparts were hindered for the same reason^(^ ^42^ ^)^.

Of the other predictors of stunting, male children were significantly more likely to be stunted in all four surveys, matching the finding in ten sub-Saharan African countries where male children consistently had a higher frequency of stunting^(^ ^43^ ^)^. Previous epidemiological studies in neonatology found morbidity and mortality to be higher in male infants and children than female counterparts, suggesting that boys are generally more vulnerable^(^ ^44^ ^)^. Similar to results from Congo, thin mothers were most likely to have stunted children, while overweight mothers were least likely^(^ ^29^ ^)^. When compared with children less than 12 months of age, older children were significantly more likely to be stunted, consistent with findings in Nepal and Burundi^(^ ^2^ ^,^ ^40^ ^)^. The OR was highest for children between 24 and 35 months of age. It is not surprising that the stunting rate increases with age (at least up to 3 years) as the growth deficit is cumulative and there is limited catch-up growth^(^ ^45^ ^)^. Other possible explanations are prolonged breast-feeding into the second year of life, early or late introduction of complementary foods, and insufficient frequency of feeding^(^ ^34^ ^)^. However, children aged 36–59 months were less likely to be stunted than those 24–35 months old, and the reasons are unclear. It is possible some of them had recovered from stunting^(^ ^46^ ^,^ ^47^ ^)^ or the severely stunted ones had died, leaving the less stunted ones to survive beyond 2 years of age. Children with small birth size were more likely to be stunted than those with normal size since birth size is associated with birth height, which is a predictor of height at older ages^(^ ^48^ ^)^. Morbidity, including diarrhoea, was associated with increased chance of being stunted, although this may be reverse causality because stunted children are at a greater risk of diarrhoea. Both birth size and morbidity showed similar impact on childhood malnutrition in Kenya and Nigeria^(^ ^6^ ^,^ ^32^ ^)^. Considering all these predictors, low-birth-size Ugandan boys in their third year of life are at the highest risk of stunting. This risk is even higher following bouts of diarrhoea. Infant girls with normal birth size are at the lowest risk of stunting. Teen mothers were more likely to have stunted children. Younger women tend to have less child-rearing knowledge or experience; in addition, they may have not finished secondary education.

According to Fig. 1, the trends of stunting from the NCHS/CDC reference and the WHO growth standards are parallel, with the WHO standards-based trend higher than the NCHS/CDC reference-based trend, suggesting that the NCHS/CDC reference underestimated stunting among Ugandan children. Whereas using any of the two standards would be sufficient for surveillance purposes, the NCHS/CDC reference would misclassify some stunted children as normal, hindering their opportunity to receive relevant interventions. Our study demonstrates that the process of transitioning from the NCHS/CDC reference to the WHO standards not only masked a 7% decrease in stunting during the study period according to UDHS reports, but also masked the precipitous drop in 2001 and erroneously showed a moderate drop starting in 2006. According to our study, it appears that interventions implemented in the 1990s rather than 2000s may be responsible for most of the observed decease in stunting. Whether the stunting reduction mask related to transitioning between the NCHS/CDC reference and WHO growth standards observed in our study explains the minimal change in prevalence in stunting reported in other studies needs further investigation^(^ ^27^ ^,^ ^33^ ^)^.

There are strengths and limitations in the present study. Strengths include large sample size for all surveys, thereby allowing the use of a comprehensive set of variables, and four surveys to analyse trends. We believe that our findings are robust as there are no major differences whether we use continuous HAZ or the binary stunting variable, severe or moderate stunting, or the NCHS/CDC reference or WHO standards to evaluate predictors of stunting. Limitations are that cross-sectional analysis did not allow following individual children as they grew to enable us to establish causality between the predictor variables and stunting. Inconsistent breast-feeding information across the surveys did not allow meaningful analysis of this important factor. Change in the number of regions between surveys forced us to use the broadest regions used in earlier surveys. Availability of complete data varied from survey to survey, and the number of under-5 children per household dwindled with subsequent surveys; the latter for a good reason though, namely fewer children per household due to decreasing fertility rate that dropped from 7 to below 6 children per woman during the study period^(^ ^49^ ^)^. The smaller numbers in the 2006 and 2011 surveys may have compromised the power to detect associations in those surveys compared with the 1995 and 2001 surveys, although we did not find many differences across surveys apart from the wealth index variable.

## Conclusions

The aims of the present study were to describe the trend of under-5 childhood stunting over 16 years in Uganda and to analyse its association with region, wealth and maternal education. At the national level, the analysis showed that under-5 childhood stunting has been declining. But not all regions or groups of children benefited equally from the decline. As of 2011, children living in the Western region, living in middle-income households and with uneducated mothers were the most vulnerable to stunting. In addition, despite the general decrease, the prevalence of stunting remains high. Policies and practices need to be put in place to address this high level of stunting, guided by demographic details.

## References

[1] World Health Organization (2016) Nutrition | Stunting in a nutshell. http://www.who.int/nutrition/healthygrowthproj_stunted_videos/en/ (accessed June 2018).

[2] Tiwari R, Ausman LM & Agho KE (2014) Determinants of stunting and severe stunting among under-fives: evidence from the 2011 Nepal Demographic and Health Survey. BMC Pediatr 14, 239. 25262003 10.1186/1471-2431-14-239 PMC4263111

[3] de Onis M, Blössner M & Borghi E (2012) Prevalence and trends of stunting among pre-school children, 1990–2020. Public Health Nutr 15, 142–148. 21752311 10.1017/S1368980011001315

[4] Uganda Bureau of Statistics & ICF International, Inc. (2012) Uganda Demographic and Health Survey 2011. https://dhsprogram.com/pubs/pdf/fr264/fr264.pdf (accessed June 2018).

[5] Muhoozi GKM, Atukunda P, Mwadime R et al. (2016) Nutritional and developmental status among 6- to 8-month-old children in southwestern Uganda: a cross-sectional study. Food Nutr Res 60, 30270. 27238555 10.3402/fnr.v60.30270 PMC4884678

[6] Masibo PK & Makoka D (2012) Trends and determinants of undernutrition among young Kenyan children: Kenya Demographic and Health Survey; 1993, 1998, 2003 and 2008–2009. Public Health Nutr 15, 1715–1727. 22694984 10.1017/S1368980012002856

[7] Habaasa G (2015) An investigation on factors associated with malnutrition among underfive children in Nakaseke and Nakasongola districts, Uganda. BMC Pediatr 15, 134. 26403539 10.1186/s12887-015-0448-y PMC4582820

[8] Willey BA, Cameron N, Norris SA et al. (2009) Socio-economic predictors of stunting in preschool children – a population-based study from Johannesburg and Soweto. S Afr Med J 99, 450–456. 19736848

[9] Kikafunda JK, Walker AF, Collett D et al. (1998) Risk factors for early childhood malnutrition in Uganda. Pediatrics 102, e45. 9755282 10.1542/peds.102.4.e45

[10] Fotso J-C (2006) Child health inequities in developing countries: differences across urban and rural areas. Int J Equity Health 5, 9. 16831231 10.1186/1475-9276-5-9 PMC1544325

[11] Ministry of Finance Planning and Economic Development, Uganda (2013) Millennium Development Goals Report for Uganda. Special Theme: Drivers of MDG Progress in Uganda and the Implications for the Post-2015 Development Agenda. http://www.ug.undp.org/content/dam/uganda/docs/UNDPUg-2013MDGProgress Report-Oct 2013.pdf (accessed June 2018).

[12] The World Bank (2016) GDP per capita (current US$). https://data.worldbank.org/indicator/NY.GDP.PCAP.CD?locations=UG (accessed June 2018).

[13] Statistics Department Uganda & Macro International, Inc. (1996) Uganda Demographic and Health Survey 1995. https://www.dhsprogram.com/pubs/pdf/FR69/FR69.pdf (accessed June 2018).

[14] The World Bank (2005) Uganda’s Nutrition and Early Child Development Project – Counting on Communication. http://documents.worldbank.org/curated/en/715231468310726837/pdf/331760infob111.pdf (accessed June 2018).

[15] UNICEF Uganda (2018) Keeping Children Alive, Safe and Learning. https://www.unicef.org/uganda/UNICEF_Uganda_Keeping_Children_Alive_Safe_and_Learning_new_version(1).pdf (accessed June 2018).

[16] UNICEF Uganda (2018) Uganda. https://www.unicef.org/uganda/alive.html (accessed June 2018).

[17] US Agency for International Development (2015) Uganda: Nutrition Profile. https://www.usaid.gov/what-we-do/global-health/nutrition/countries/uganda-nutrition-profile (accessed June 2018).

[18] Ministry of Health Uganda (2005) Health Sector Strategic Plan II 2005/06–2009/2010. http://siteresources.worldbank.org/INTPRS1/Resources/383606-1201883571938/Uganda_HSSP_2.pdf (accessed June 2018).

[19] Uganda Bureau of Statistics & ORC Macro (2000) Uganda Demographic and Health Survey 2000–2001. https://www.ubos.org/wp-content/uploads/publications/03_2018Uganda_DHS_2000-01_Final_Report.pdf (accessed June 2018).

[20] Uganda Ministry of Health & Macro International, Inc. (2007) Uganda Demographic and Health Survey 2006. https://www.dhsprogram.com/pubs/pdf/FR194/FR194.pdf (accessed June 2018).

[21] Murthy GVS, Fox S, Sivasubramaniam S et al. (2013) Prevalence and risk factors for hypertension and association with ethnicity in Nigeria: results from a national survey. Cardiovasc J Afr 24, 233. 10.5830/CVJA-2013-058 PMC3896106 24042732

[22] Frankel MR, McNaghten A, Shapiro MF et al. (2012) A probability sample for monitoring the HIV-infected population in care in the US and in selected states. Open AIDS J 6, 67–76. 23049655 10.2174/1874613601206010067 PMC3462615

[23] WHO Multicentre Growth Reference Study Group (2006) WHO Child Growth Standards based on length/height, weight and age. Acta Paediatr Suppl 450, 76–85. 16817681 10.1111/j.1651-2227.2006.tb02378.x

[24] Centers for Disease Control and Prevention, National Center for Health Statistics (2000) CDC Growth Charts. https://www.cdc.gov/growthcharts/cdc_charts.htm (accessed June 2018).

[25] Alemu T & Umeta M (2016) Prevalence and predictors of ‘small size’ babies in Ethiopia: in-depth analysis of the Ethiopian Demographic and Health Survey, 2011. Ethiop J Health Sci 26, 243–250. 27358545 10.4314/ejhs.v26i3.7 PMC4913192

[26] Dahlui M, Azahar N, Oche OM et al. (2016) Risk factors for low birth weight in Nigeria: evidence from the 2013 Nigeria Demographic and Health Survey. Glob Health Action 9, 28822. 26790460 10.3402/gha.v9.28822 PMC4720686

[27] Barankanira E, Molinari N, Msellati P et al. (2017) Stunting among children under 3 years of age in Côte d’Ivoire: spatial and temporal variations between 1994 and 2011. Public Health Nutr 20,1627–1639. 28367794 10.1017/S1368980017000544 PMC10261337

[28] Vonaesch P, Tondeur L, Breurec S et al. (2017) Factors associated with stunting in healthy children aged 5 years and less living in Bangui (RCA). PLoS One 12, e0182363. 28796794 10.1371/journal.pone.0182363 PMC5552116

[29] Kismul H, Acharya P, Mapatano MA et al. (2018) Determinants of childhood stunting in the Democratic Republic of Congo: further analysis of Demographic and Health Survey 2013–14. BMC Public Health 18, 74. 10.1186/s12889-017-4621-0 PMC5540220 28764669

[30] Hagos S, Hailemariam D, WoldeHanna T et al. (2017) Spatial heterogeneity and risk factors for stunting among children under age five in Ethiopia: a Bayesian geo-statistical model. PLoS One 12, e0170785. 28170407 10.1371/journal.pone.0170785 PMC5295674

[31] Chirande L, Charwe D, Mbwana H et al. (2015) Determinants of stunting and severe stunting among under-fives in Tanzania: evidence from the 2010 cross-sectional household survey. BMC Pediatr 15, 165. 26489405 10.1186/s12887-015-0482-9 PMC4618754

[32] Akombi BJ, Agho KE, Hall JJ et al. (2017) Stunting and severe stunting among children under-5 years in Nigeria: a multilevel analysis. BMC Pediatr 17, 15. 28086835 10.1186/s12887-016-0770-z PMC5237247

[33] Hoffman D, Cacciola T, Barrios P et al. (2017) Temporal changes and determinants of childhood nutritional status in Kenya and Zambia. J Health Popul Nutr 36, 27. 28583185 10.1186/s41043-017-0095-z PMC5460439

[34] Wamani H, Åstrøm AN, Peterson S et al. (2005) Infant and young child feeding in Western Uganda: knowledge, practices and socio-economic correlates. J Trop Pediatr 51, 356–361. 15947011 10.1093/tropej/fmi048

[35] Biondi D, Kipp W, Jhangri GS et al. (2011) Risk factors and trends in childhood stunting in a district in Western Uganda. J Trop Pediatr 57, 24–33. 20554516 10.1093/tropej/fmq043

[36] Goode PM (1989) *Edible Plants of Uganda: The Value of Wild and Cultivated Plants as Food. FAO Food and Nutrition Paper* no. 42. Rome: FAO.

[37] Whyte MA & Kyaddondo D (2006) ‘We are not eating our own food here’: food security and the cash economy in eastern Uganda. Land Degrad Dev 17, 173–182.

[38] Egede LE, Voronca D, Walker RJ et al. (2017) Rural–urban differences in trends in the wealth index in Kenya: 1993–2009. Ann Glob Health 83, 248–258. 28619399 10.1016/j.aogh.2017.04.001 PMC5487220

[39] Steinert JI, Cluver LD, Melendez-Torres GJ et al. (2018) One size fits all? The validity of a composite poverty index across urban and rural households in South Africa. Soc Indic Res 136, 51–72. 29497232 10.1007/s11205-016-1540-x PMC5816112

[40] Nkurunziza S, Meessen B, Van Geertruyden JP et al. (2017) Determinants of stunting and severe stunting among Burundian children aged 6–23 months: evidence from a national cross-sectional household survey, 2014. BMC Pediatr 17, 176. 28743238 10.1186/s12887-017-0929-2 PMC5526249

[41] Keino S, Plasqui G, Ettyang G et al. (2014) Determinants of stunting and overweight among young children and adolescents in sub-Saharan Africa. Food Nutr Bull 35, 167–178. 25076764 10.1177/156482651403500203

[42] Ugandan Bureau of Statistics (2012) Education Sector: Gender Statistics Profile. https://www.ubos.org/wp-content/uploads/publications/04_2018Education_Sector_Gender_Statistics_Profile.pdf (accessed June 2018).

[43] Wamani H, Åstrøm AN, Peterson S et al. (2007) Boys are more stunted than girls in Sub-Saharan Africa: a meta-analysis of 16 demographic and health surveys. BMC Pediatr 7, 17. 17425787 10.1186/1471-2431-7-17 PMC1865375

[44] Pollak A & Birnbacher R (2004) Preterm male infants need more initial respiratory support than female infants. Acta Paediatr 93, 447–448. 15188966 10.1080/08035250410025744

[45] Georgiadis A & Penny ME (2017) Child undernutrition: opportunities beyond the first 1000 days. Lancet Public Health 2, e399. 29253410 10.1016/S2468-2667(17)30154-8

[46] Prentice AM, Ward KA, Goldberg GR et al. (2013) Critical windows for nutritional interventions against stunting. Am J Clin Nutr 97, 911–918. 23553163 10.3945/ajcn.112.052332 PMC3628381

[47] Georgiadis A, Benny L, Galab S et al. (2017) Growth recovery and faltering through early adolescence in low- and middle-income countries: determinants and implications for cognitive development. Soc Sci Med 179, 81–90. 28260638 10.1016/j.socscimed.2017.02.031 PMC5380196

[48] Eide MG, Øyen N, Skjœrven R et al. (2005) Size at birth and gestational age as predictors of adult height and weight. Epidemiology 16, 175–181. 15703531 10.1097/01.ede.0000152524.89074.bf

[49] Blacker J, Opiyo C, Jasseh M et al. (2005) Fertility in Kenya and Uganda: a comparative study of trends and determinants. Popul Stud 59, 355–373. 10.1080/00324720500281672 16249155

