# Supplementary material for: Trends and determinants of stunting among under-5s: evidence from the 1995, 2001, 2006 and 2011 Uganda Demographic and Health Surveys
Source: Public Health Nutr. 2018 Aug 29;21(16):2915–28. doi: 10.1017/S1368980018001982 (PMC6190071; doi:10.1017/S1368980018001982)
Supplement: Supplementary file 1 [file S1368980018001982sup001.docx]

**Supplementary material**

**Supplemental Table 1: Items used for creating the wealth index**

| Communication | Radio^+, ++, +++^  Television^+, ++, +++^  Telephone ^+, +++^  Mobile telephone^++, +++^  Watch^+++^ |
| --- | --- |
| Transport | Bicycle ^+, ++, +++^  Motorcycle ^+, ++, +++^  Car/truck^+, ++, +++^  Boat/ Canoe^+, ++, +++^  Boat with a motor^++, +++^  Donkey^+^  Animal drawn cart^++, +++^ |
| Health and Hygiene | Piped drinking water in residence^+, ++, +++^  Piped drinking water into yard^++, +++^  Piped drinking water from public tap ^+, ++, +++^  Piped drinking water outside of residence^+^  Open well drinking water in yard/plot^+, ++^  Open well drinking water outside yard^+, +++^  Protected well drinking water in yard^+, ++, +++^  Unprotected well drinking water^++, +++^  Protected well drinking water outside yard^+, +++^  Borehole well drinking water in yard^+, +++^  Borehole well drinking water outside yard^+, ++, +++^  Drinking water from spring^+, +++^  Uses river, canal, or surface water for drinking^+, +++^  Uses rain for drinking water^+, ++, +++^  Uses tanker truck for drinking water^+, ++, +++^  Uses bottled water for drinking^+, ++, +++^  Drinking water from gravity flow scheme^+^  Own flushing toilet^+, ++, +++^  Shared flushing toilet^+, ++, +++^  Has traditional pit latrine^+,^  Covered pit latrine with no slab^++, +++^  Covered pit latrine with slab^++, +++^  Has private pit latrine^++^  Shares pit latrine^++^  Uncovered pit latrine with slab^+++^  Composting toilet/ecosan^+++^  Ecosan toilet^+++^  Has VIP latrine^+, ++, +++^  Uses shared VIP latrine^++, +++^  Uses bush or field as latrine^+, ++, +++^  Other type of latrine^+, +++^ |
| Household essentials | Has electricity^+, ++, +++^  Lantern^+^  Cupboard^+, ++, +++^  Table^++, +++^  Chairs^++, +++^  Sofa set^++, +++^  Bed^++, +++^  Clock^++, +++^  Has refrigerator^+, ++, +++^  Has video recorder  Has cassette player^++, +++^  Has dirt, earth, dung principal floor surface^+, ++, +++^  Has parquet principal floors^+, ++, +++^  Has vinyl or asphalt tile principal floors^+^  Has ceramic tile principal floors^+, +++^  Has cement principal floor^+, ++, +++^  Has brick floor^+++^  Has stone floor^+++^  Has other type of flooring^+, +++^  Uses electricity as cooking fuel^+, ++, +++^  Uses gas as cooking fuel^+, ++, +++^  Uses biogas as cooking fuel^+, +++^  Uses kerosene as cooking fuel^+, ++, +++^  Uses coal as cooking fuel^+^  Uses charcoal as cooking fuel^+, ++, +++^  Uses wood/straw as cooking fuel^+, ++, +++^  Does not cook^+++^  Uses other type of cooking^+, ++, +++^  Has thatch for walls^+, ++, +++^  Walls of mud and poles^+, ++, +++^  Walls of unburnt brick^+, ++, +++^  Walls of unburnt brick and plaster^+++^  Walls of burnt bricks and mud^+, ++, +++^  Walls of burnt bricks and cement^+, ++, +++^  Wood timber for walls^+, ++, +++^  Has bricks, cement blocks, concrete walls^+, +++^  Has stone walls^+, +++^  Has thatched roofing^+, +++^  Has wood/planks roofing^+++^  Has corrugated iron roofing^+, ++, +++^  Has asbestos roofing^+++^  Has tile roof ^+++^  Has tin roof ^+++^  Has cement roof^++^  Has nat roof ^++^  Has finished roof ^++^  Uses electricity for lighting^+^  Uses biogas for lighting^+^  Uses kerosene for lighting^+^  Uses charcoal for lighting^+^  Uses dung for lighting^+^ |
| Household members | Number of household members per sleeping room^++, +++^ |
| Household farming assets | If household works own family agriculture land^++^  Cattle own^++^  Cows, bulls own^++^  Horses, donkeys, mules own^++^  Goats own^++^  Sheep own^++^  Chickens own^++^ |

+ Included in 2001 survey

++ Included in 2006 survey

+++ Included in 2011 survey

**Supplemental Table 2: Analysis of sub-regions in the overall multivariable model for moderate-severe stunting (primary analysis) for the 2006 and 2011 surveys**

| Variable: regions | Moderate-severe stunting, N=4,433, OR (95% CI) |
| --- | --- |
| Eastern (ref.)  Central 1  Central 2  Kampala  East Central  North  West Nile  Western  Southwest | 1.00  1.15 (0.79, 1.66)  1.24 (0.88, 1.76)  1.69 (1.14, 2.50)***  1.42 (1.06, 1.90)**  1.25 (0.94, 1.66)  1.26 (0.93, 1.73)  1.65 (1.22 2.23)***  2.23 (1.63, 3.06)*** |

ref., reference category.

*P ≤ 0.05, **P ≤ 0.01, ***P ≤ 0.001

Model is adjusted for survey year indicator, household, maternal, and child-related variables.

**Supplemental Table 3: Analysis of discrete years of education in the overall multivariable model for moderate-severe stunting**

| Variable: continuous years of education | moderate-severe stunting, N=**14,747**, OR (95% CI) |
| --- | --- |
| 0 (ref.)  1  2  3  4  5  6  7  8  9  10  11  12 | 1.00  1.01 (0.82, 1.26)  1.00 (0.85, 1.18)  0.96 (0.82, 1,12)  0.90 (0.77, 1.05)  0.91 (0.78, 1.06)  0.84 (0.72, 0.97)*  0.63 (0.54, 0.74)***  0.71 (0.54, 0.94)*  0.88 (0.66, 1.16)  0.73 (0.53, 1.01)  0.63 (0.46, 0.86)**  0.43 (0.30, 0.61)*** |

ref., reference category.

*P ≤ 0.05, **P ≤ 0.01, ***P ≤ 0.001

Model is adjusted for survey year indicator, household, maternal, child-related variables.

**Supplemental Table 4: Overall WHO standard-based multivariable models for moderate-severe stunting (primary analysis), severe stunting, weight z-score, and CDC standard- based moderate-severe stunting**

| **Variable** | **Moderate-Severe Stunting, N=14,747** | **Severe Stunting N=14,747** | **Continuous Z-Score N=14,747** | **Moderate-Severe Stunting Using CDC Standard, N=14,747** |
| --- | --- | --- | --- | --- |
|  | OR (95% CI) | OR (95% CI) | β (Standard Error) | OR (95% CI) |
| Intercept | - | - | 0.1218 (0.07) |  |
| **Household characteristics** | | | |  |
| Region  Central  Northern  Western  Eastern (ref.) | 1.25 (1.11, 1.42)***  1.02 (0.89, 1.16)  1.74 (1.55, 1.96)***  1.00 | 1.26 (1.07, 1.48)**  1.16 (0.98, 1.37)  1.80 (1.56, 2.08)***  1.00 | -0.19 (0.04)***  -0.07 (0.04)  -0.41 (0.04)***  0.00 | 1.29 (1.13, 1.46)***  1.06 (0.92, 1.21)*  1.77 (1.57, 1.99)***  1.00 |
| Residence  Rural  Urban (ref.) | 1.20 (1.03, 1.40)*  1.00 | 1.26 (1.02, 1.56)*  1.00 | -0.19 (0.05)***  0.00 | 1.22 (1.04, 1.43)*  1.00 |
| Wealth Index  Poorest  Poorer  Middle  Richer  Richest (ref.) | 1.73 (1.45, 2.06)***  1.50 (1.26, 1.78)***  1.61 (1.36, 1.90)***  1.37 (1.16, 1.61)***  1.00 | 1.84 (1.45, 2.34)***  1.70 (1.35, 2.15)***  1.71 (1.36, 2.14)***  1.46 (1.16, 1.83)**  1.00 | -0.37 (0.05)***  -0.30 (0.05)***  -0.34 (0.05)***  -0.20 (0.05)***  0.00 | 1.67 (1.40, 2.01)***  1.54 (1.29, 1.84)***  1.67 (1.41, 1.98)***  1.39 (1.18, 1.65)***  1.00 |
| Source of drinking water  Non-improved  Improved (ref.) | 1.09 (1.00, 1.18)  1.00 | 1.03 (0.92, 1.14)  1.00 | -0.01 (0.03)  0.00 | 1.06 (0.97, 1.16)  1.00 |
| **Maternal characteristics** | | | |  |
| Age  15-19  20-30  31-49 (ref.) | 1.57 (1.31, 1.87)***  1.18 (1.08, 1.29)***  1.00 | 1.44 (1.15, 1.81)**  1.11 (0.99, 1.24)  1.00 | -0.27 (0.06)***  -0.10 (0.03)***  0.00 | 1.52 (1.27, 1.83)***  1.19 (1.08, 1.31)***  1.00 |
| BMI  Pregnant/postpartum  Thin  Over/Obese  Normal (ref.) | 1.04 (0.94, 1.16)  1.08 (0.92, 1.27)  0.70 (0.61, 0.79)***  1.00 | 1.04 (0.91, 1.19)  1.15 (0.94, 1.41)  0.70 (0.58, 0.84)***  1.00 | -0.01 (0.03)  -0.10 (0.06)  0.25 (0.04)***  0.00 | 1.04 (0.93, 1.15)  1.11 (0.94, 1.31)  0.71 (0.62, 0.81)***  1.00 |
| Highest educational level  None  Primary  Secondary+ (ref.) | 1.34 (1.14, 1.59)***  1.20 (1.04, 1.38)*  1.00 | 1.37 (1.08, 1.73)**  1.25 (1.00, 1.55)*  1.00 | -0.11 (0.05)*  -0.10 (0.04)*  0.00 | 1.52 (1.28, 1.81)***  1.32 (1.13, 1.54)***  1.00 |
| Work Status  Unemployed  Self-employed  Employed (ref.) | 1.06 (0.92, 1.23)  1.18 (1.04, 1.34)*  1.00 | 1.21 (0.99, 1.47)  1.26 (1.06, 1.51)**  1.00 | -0.08 (0.05)  -0.14 (0.04)***  0.00 | 1.01 (0.88, 1.17)  1.07 (0.94, 1.23)  1.00 |
| **Child-related characteristics** | | | |  |
| Sex  Male  Female (ref.) | 1.42 (1.31, 1.54)***  1.00 | 1.47 (1.32, 1.64)***  1.00 | -0.22 (0.03)***  0.00 | 1.26 (1.15, 1.37)***  1.00 |
| Current age in months  13-23  24-35  36-59  0-12 (ref.) | 3.22 (2.85, 3.65)***  4.61 (4.05, 5.25)***  3.75 (3.32, 4.23)***  1.00 | 3.64 (3.04, 4.37)***  5.38 (4.47, 6.49)***  4.05 (3.38, 4.86)***  1.00 | -0.92 (0.04)***  -1.20 (0.04)***  -1.10 (0.04)***  0.00 | 4.11 (3.61, 4/69)***  3.76 (3.27, 4.32)***  4.40 (3.86, 5.01)***  1.00 |
| Low birth size  Yes  No (ref.) | 1.59 (1.44, 1.77)***  1.00 | 1.56 (1.37, 1.77)***  1.00 | -0.35 (0.03)***  0.00 | 1.56 (1.40, 1.73)***  1.00 |
| Preceding birth interval less than 24 months  Yes  No (ref.) | 1.19 (1.08, 1.32)***  1.00 | 1.32 (1.16, 1.49)***  1.00 | -0.12 (0.03)***  0.00 | 1.23 (1.11, 1.37)***  1.00 |
| Had fever in last two weeks  Yes  No (ref.) | 1.08 (0.99, 1.18)  1.00 | 1.01 (0.90, 1.13)  1.00 | -0.07 (0.03)*  0.00 | 1.06 (0.97, 1.16)  1.00 |
| Had diarrhea in last two weeks  Yes  No (ref.) | 1.17 (1.11, 1.23)***  1.00 | 1.16 (1.09, 1.24)***  1.00 | -0.21 (0.03)***  0.00 | 1.19, (1.12, 1.25)***  1.00 |
| Survey Year  2001  2006  2011  1995 (ref.) | 0.89 (0.81, 0.99)*  0.64 (0.57, 0.73)***  0.58 (0.51, 0.67)***  1.00 | 0.96 (0.84, 1.09)  0.64 (0.54, 0.76)***  0.67 (0.56, 0.81)***  1.00 | 0.05 (0.03)  0.34 (0.04)***  0.39 (0.04)*** | 0.94 (0.85, 1.04)  0.66 (0.58, 0.75)***  0.61 (0.53, 0.70)***  1.00 |

ref., reference category.

*P ≤ 0.05, **P ≤ 0.01, ***P ≤ 0.001

**Supplemental Fig. 1: IRB termination letter, not human subject research**


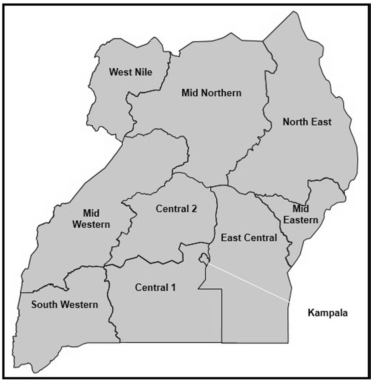


**Supplemental Fig. 2: Sub-regions of Uganda**^(41)^

Sub-regions were combined into four regions as shown below:

Northern = West Nile, Mid Northern, and North East

Eastern = East Central and Mid Eastern

Central = Central 1, Central 2, and Kampala

Western = Mid Western and South Western
